# Prevalence and clinical characteristics of incontinentia pigmenti: a nationwide population-based study

**DOI:** 10.1186/s13023-024-03480-8

**Published:** 2024-12-02

**Authors:** Laura Krogh Herlin, Sigrun Alba Johannesdottir Schmidt, Trine H. Mogensen, Mette Sommerlund

**Affiliations:** 1 Department of Dermatology, Aarhus University Hospital, Palle Juul-Jensens Boulevard 67, Aarhus N, 8200 Denmark; 2 Department of Clinical Medicine, Aarhus University, Building A, Palle Juul-Jensens Boulevard 99, Aarhus N, Denmark; 3 Department of Clinical Epidemiology, Aarhus University Hospital and Aarhus University, Olof Palmes Allé 43-45, Aarhus N, 8200 Denmark; 4 Department of Biomedicine, Aarhus University, Aarhus, Denmark; 5 Department of Infectious Diseases, Aarhus University Hospital, Palle Juul-Jensens Boulevard 67, Aarhus N, Denmark

**Keywords:** Ectodermal dysplasia, Epidemiology, IKBKG, Incontinentia pigmenti, MIM 308300, Phenotype, Prevalence

## Abstract

**Background:**

Incontinentia pigmenti (IP) is an X-linked dominant multisystemic disorder caused by pathogenic variants in the *IKBKG* gene. Population-based research into the epidemiology of IP is lacking.

**Methods:**

This nationwide cross-sectional study from Jan 1st, 1995 to August 25th, 2021, searched the Danish National Patient Registry (DNPR), the Danish National Database of Rare Genetic Diseases (RareDis) and the Danish Genodermatosis Database to identify patients recorded with a diagnosis of IP. This search was followed by diagnosis validation and collection of clinical data from patient medical records. We investigated the clinical characteristics and genetics of the final cohort of validated IP cases. We estimated the point prevalence in the Danish population, based on non-deceased IP patients currently living in Denmark. Furthermore, we estimated the birth prevalence from 1995 to 2020, assuming a diagnostic delay of up to six months.

**Results:**

We identified a validated cohort of 75 IP patients, including 71 (94.7%) females and 4 (5.3%) males. We estimated a birth prevalence of 2.37 (95% CI: 1.74–3.25) per 100,000 or 1 in 42,194. A total of 54 (72%) patients had a genetic diagnosis, including 39 (72.2%) with the recurrent exon 4–10 deletion and 10 (18.5%) with point mutations in *IKBKG*. A positive family history was reported in 53.3%. Besides the recognizable blaschkolinear skin lesions reported in 70 (93.3%) of the patients, commonly reported manifestations included the involvement of the teeth (58.7%), the central nervous system (30.7%), hair (26.7%), and eyes (22.6%), as well as nail dystrophy (16.0%).

**Conclusions:**

We identified and characterized a nationwide population-based cohort of IP patients and report a birth prevalence of 2.37 per 100,000 live births, which is twice as high as previous estimates.

## Background

**Fig. 1:**
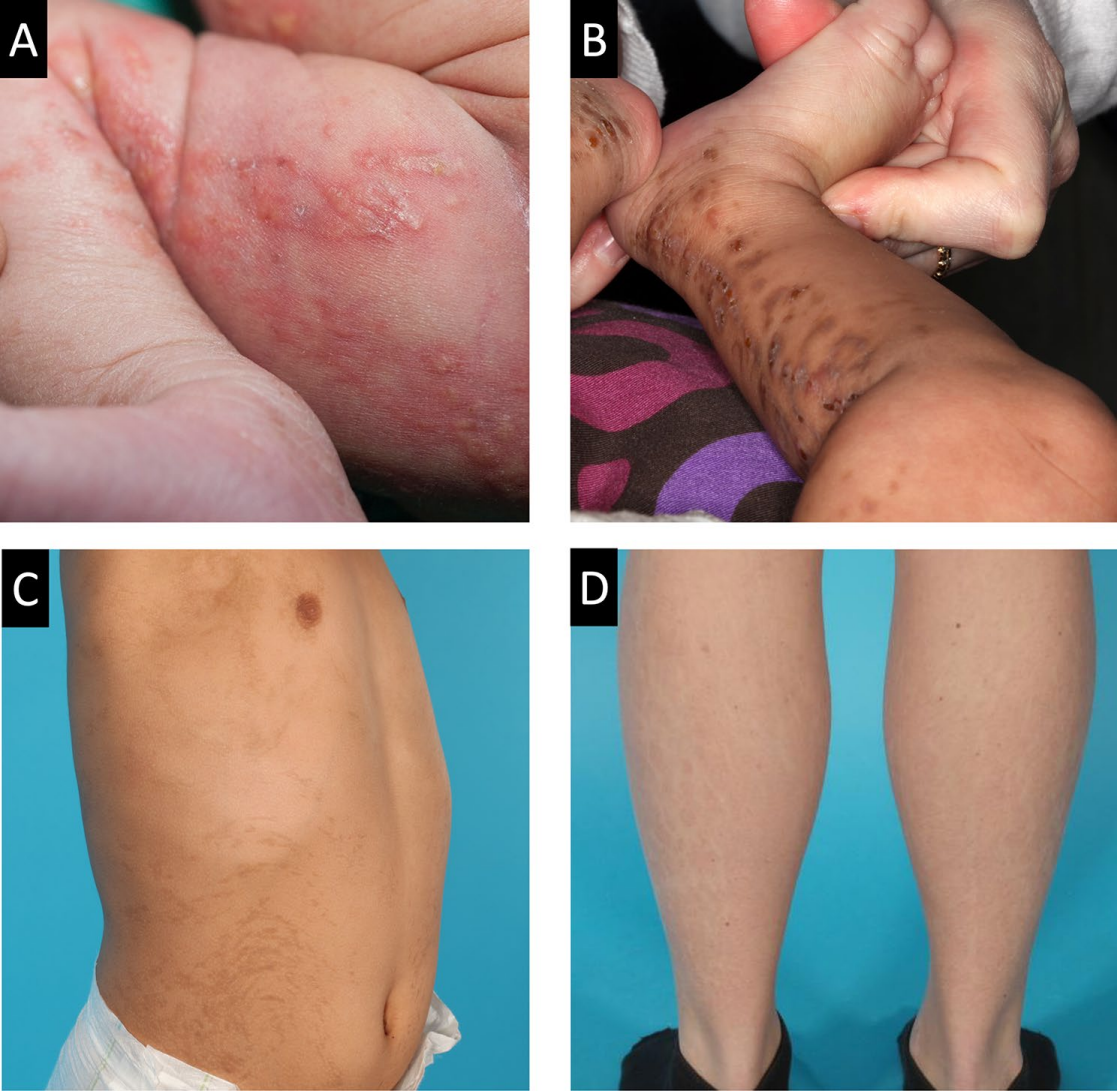
Four stages of skin lesions following Blaschko’s lines in female (**A** and **D**) and male (**B** and **C**) IP patients. **A**) Vesicular stage, **B**) verrucous hyperkeratotic stage, **C**) hyperpigmented stage, and (**D**) atrophic/hypopigmented stage

Incontinentia pigmenti (IP) (OMIM #308300), also known as Bloch-Sulzberger syndrome, is a rare genetic multisystem disorder grouped within the ectodysplasin A (EDA) pathway of ectodermal dysplasias [1, 2]. The disease primarily affects the skin, hair, nails, and teeth [3]. The skin manifestations follow the distribution of Blaschko’s lines and undergo four characteristic overlapping stages: (1) vesicular, (2) verrucous, (3) hyperpigmented, and (4) atrophic/hypopigmented (Fig. 1) [4]. In addition to the ectodermal manifestations in IP, ophthalmologic and neurologic abnormalities can be seen, partially explained by underlying endothelial inflammation and small-vessel disease [5].

IP is an X-linked dominant disorder caused by pathogenic variants in the *IKBKG* gene, which encodes NF-kappa-B essential modulator (NEMO) [6] — a regulatory protein in the NF-kappa-B signaling pathway involved in cellular proliferation, apoptosis, and inflammation [7, 8]. The pathophysiology of IP hence involves susceptibility to proapoptotic signals, which cause skin and endothelial inflammation. The latter may lead to vaso-occlusion, resulting in neurologic and ophthalmologic manifestations [5]. It is estimated that 60–80% of IP cases are caused by a recurrent deletion of exons 4–10 in the *IKBKG* gene [9, 10]. Several *IKBKG* sequence variants have also been identified as causative. The proportion of *de novo* cases is approximately 60–80% [1, 5, 11].

IP is predominantly seen in females since loss-of-function variants are lethal in male fetuses [12]. In rare cases, IP can occur in males because of postzygotic mosaicism or in combination with a 47,XXY karyotype [13, 14]. Hypomorphic variants in *IKBKG* have been reported in males, leading to the rare form of X-linked hypohidrotic ectodermal dysplasia with immunodeficiency (HED-ID) (OMIM #300291), while in females, they may cause a mild presentation of IP [15].

The birth prevalence of IP is frequently cited as 0.7 per 100.000 births based on an Orphanet report from 2013 [1, 3], increasing to 1.2 per 100,000 births in the latest 2023 series [16]. However, population-based estimates of IP occurrence are lacking, with only one study published to date. Using a nationwide Swedish hospital registry, the study estimated a period prevalence of 1 per 41,000 (equal to 2.4 per 100,000) during 2001–2020 [17]. However, the disease occurrence may have been overestimated, as the authors did not validate registered IP cases.

To improve the understanding of the epidemiology of IP, we present a nationwide and validated cohort of IP patients and provide population-based estimates of the disease prevalence and clinical characteristics.

## Methods

### Study design and setting

We performed a descriptive cross-sectional study based on a nationwide validated cohort of IP patients identified in Danish health registries from 1995 to 2021. This study followed a larger study of ectodermal dysplasias in Denmark [18].

Danish residents have access to universal tax-financed medical care [19]. Various health and social data are recorded in nationwide registries that can be linked accurately at the individual level using the unique personal identifier assigned to all Danish residents [20]. This provides a unique opportunity to conduct nationwide epidemiologic studies.

### Data sources and patient identification

We searched for patients registered with IP in the Danish National Patient Registry (DNPR), which records detailed information on hospital care for all Danish residents, including data from all admissions since 1977 and outpatient visits since 1995 [21]. Diagnoses are registered using the International Classification of Diseases (ICD), version 8 until the end of 1993 and version 10 thereafter [21]. From 1995 to 2021, we searched for patients registered with the specific ICD-10 code for IP (Q82.3) to identify cases with a putative diagnosis.

Additionally, we searched the Danish National Database of Rare Genetic Diseases (RareDis) and the Danish Genodermatosis Database for cases registered with an ICD-10 (Q82.3), OMIM (#308300), or Orphanet (#464) code for IP. RareDis is a national clinical database encompassing individual-level clinical and genetic data on patients with rare diseases registered by treating clinicians since 2007. The Danish Genodermatosis Database includes clinical and diagnostic information, as well as family-case linkage on Danish patients with genodermatoses from the five departments of dermatology in Denmark since 2014.

### Patient validation and characterization

One author (LKH) reviewed the identified patients’ medical records to validate diagnoses and collect relevant clinical data, including patient/family history, phenotypical features, patient course and management, and genetic test results, to the extent available. Data were extracted to a prepared REDCap form as previously described [18]. As the reference standard for validation, we applied the updated diagnostic IP criteria from a European network published in 2020 [3], thus including all genetically and/or clinically confirmed IP patients in our validated cohort. In familial cases, all patients were evaluated individually. However, patients with a positive family history only required one minor criterion to establish the diagnosis [3].

### Statistical analyses

Demographic and clinical data were summarized as frequencies with percentages for categorical variables and medians with interquartile ranges (IQRs) for continuous variables. Small numbers < 3 were obscured due to ethical considerations.

We calculated the positive predictive value (PPV) with 95% confidence intervals (CI) as the number of patients with a validated IP diagnosis, divided by those registered in DNPR with Q82.3. To examine the occurrence of IP, we estimated the point prevalence with 95% CI as the number of validated non-deceased/non-emigrated IP patients divided by the number of Danish residents (*N* = 5,850,189) reported by Statistics Denmark at the start of the third quarter of 2021 (https://www.statistikbanken.dk, date of access January 30th, 2024). We also estimated the birth prevalence from the number of patients born from 1995 to 2020 divided by the number of live births, assuming a diagnostic delay before receiving a first-time diagnosis of up to six months. This assumption was supported by an upper quartile of the age at diagnosis of 11 weeks for patients born since the study commencement in 1995. We performed prevalence calculations for both sexes combined and for females only.

All analyses were performed using Stata (v18.0; StataCorp. LP, College Station, TX, USA), and we adhered to the STROBE Reporting Guidelines.

## Results

### Cohort identification

**Fig. 2:**
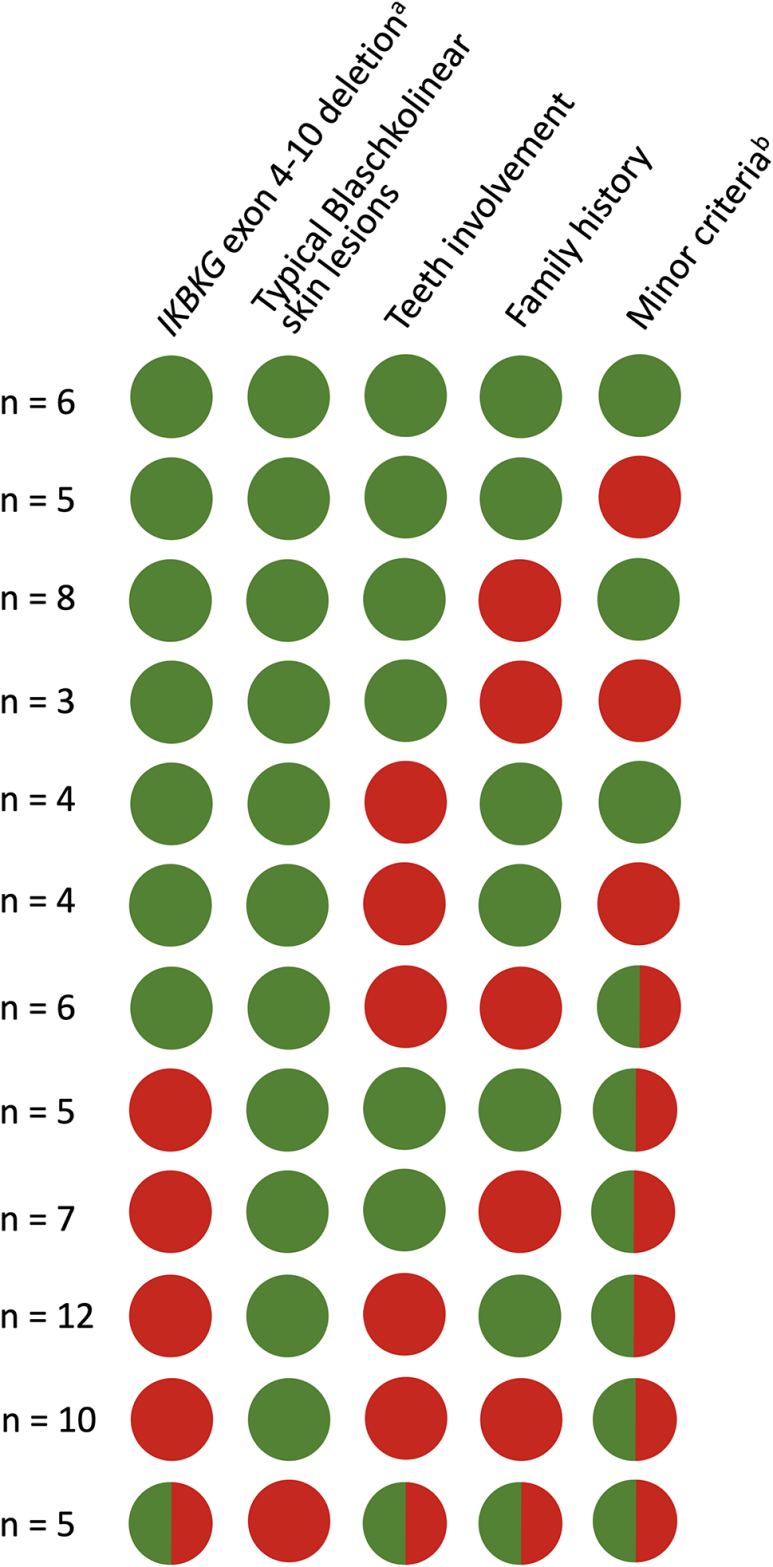
Diagnostic criteria fulfilled among confirmed cases. Diagnostic criteria according to Bodemer et al. [3]. Major criteria include identification of *IKBKG* exon 4–10 deletion (^a^other pathogenic variants are not included in the diagnostic criteria), typical Blaschkolinear lesions, and teeth abnormalities, and only one major criterion is needed to establish the diagnosis. ^b^Minor criteria include eosinophilia, hair involvement, nail involvement, mammary gland involvement, characteristic skin histology, and retinal disease. In the presence of positive family history, only one minor criterion is required for the diagnosis. Green = criterion met, red = criterion not met. Two-colored circles indicate that within a group, some patients met the criterion and some did not, and these are used when further specification would identify small groups below three individuals

From the DNPR search, we identified a total of 107 people registered with Q82.3 during the study period. Ninety-seven (90.7%) records were available for validation, of which 71 were confirmed with IP (positive predictive value: 73%, 95% CI: 63-81%). Searching clinical databases (RareDis/Danish Genodermatosis Database), an additional four patients were identified, resulting in a validated cohort of 75 IP patients included in the study, all fulfilling the pre-specified diagnostic criteria. The validation and diagnostic criteria met among confirmed cases are illustrated in Fig. 2.

Of the 26 patients from the DNPR search excluded following validation, 15 were coding mistakes, where the appropriate diagnosis code would have been Q82.5 (congenital non-neoplastic nevus) or Q28.3 (other malformations of cerebral vessels) in several of the cases. Four patients were suspected of IP but received other diagnoses later (e.g., pigment mosaicism). The remaining seven cases were excluded for other reasons (e.g., did not fulfill clinical criteria or received the diagnosis code only because of family history). Two had other ectodermal dysplasia diseases.

The validated IP patients included 71 (94.7%) females and 4 (5.3%) males (Table 1). The patients had visited various departments related to their diagnosis, including dermatology (*n* = 50, 66.7%), pediatrics (*n* = 25, 33.3%), odontology (*n* = 31, 41.3%), genetics (*n* = 27, 36%), specialized centers of rare diseases (*n* = 17, 22.7%), ophthalmology (*n* = 18 (24%), and neurology (*n* = 4, 5.3%).

**Table 1:** Demographic, diagnostic, and reported clinical characteristics of 75 patients with a validated diagnosis of IP, Denmark, 1995–2021^a^

Variable	*n*	%
Sex		
Female	71	95.0%
Male	4	5.0%
Positive family history	40	53.3%
*De novo* cases	35	46.7%
Median age (IQR) at diagnosis, years	3	(0–29)
Median age (IQR) at genetic diagnosis, years	18	(1–31)
Histopathological IP findings	6	8.0%
Genetic diagnosis (see Fig. 3)	54	72.0%
CNS involvement	23	30.7%
Seizures	10	13.3%
Spastic paresis/motor impairment	7	9.3%
Ischemic strokes	6	8.0%
Cognitive impairment	6	8.0%
Ophthalmologic involvement	17	22.6%
Retinal vasculopathy	11	14.7%
Visual loss	9	12.0%
Strabismus	5	6.7%
Eye malformation	< 3	
Cataract	< 3	
Hair involvement	20	26.7%
Hypotrichosis/focal alopecia	16	21.3%
Wooly texture of hair	4	5.3%
Brittle hair	< 3	
Dental involvement	44	58.7%
Hypo-/oligodontia	39	52.0%
Cone-shaped teeth	15	20.0%
Delayed eruption	3	4.0%
Skin involvement with blaschkolinear IP lesions	70	93.3%
Nail involvement		
Nail dystrophy	12	16.0%
Anonychia	< 3	
Other features		
Atopic comorbidity	15	20.0%
History of miscarriage (in females)	11	15.5%
Recurrent infections	9	12.0%
Limb defects	3	4.0%

Abbreviations CNS, Central nervous system; IP, Incontinentia pigmenti; IQR, Interquartile range

^a^ Small numbers (< 3) are excluded from totals when required to obscure the values

### Prevalence of incontinentia pigmenti

In total, 71 of 5,850,189 Danish residents were alive and living with a diagnosis of IP on July 1st 2021 (excluding four IP patients who had died or emigrated), yielding a point prevalence of 1.21 per 100,000 (95% CI: 0.96–1.53 per 100,00), equal to 1 in 82,397 people. The point prevalence among females was 2.28 per 100,000 (95% CI: 1.79–2.89 per 100,000) or 1 in 43,860 females.

From 1995 to 2020, 39 cases were diagnosed among 1,642,344 live births at a birth prevalence of 2.37 per 100,000 (95% CI: 1.74–3.25 per 100,000) or 1 in 42,111 (95% CI: 1 per 30,807 − 57,563). The birth prevalence was highest in females, with 36 cases detected among 799,487 live births, corresponding to 4.5 per 100,000 births (95% CI: 3.25–6.23 per 100,000) or 1 in 22,222.

### Genetic findings

**Fig. 3:**
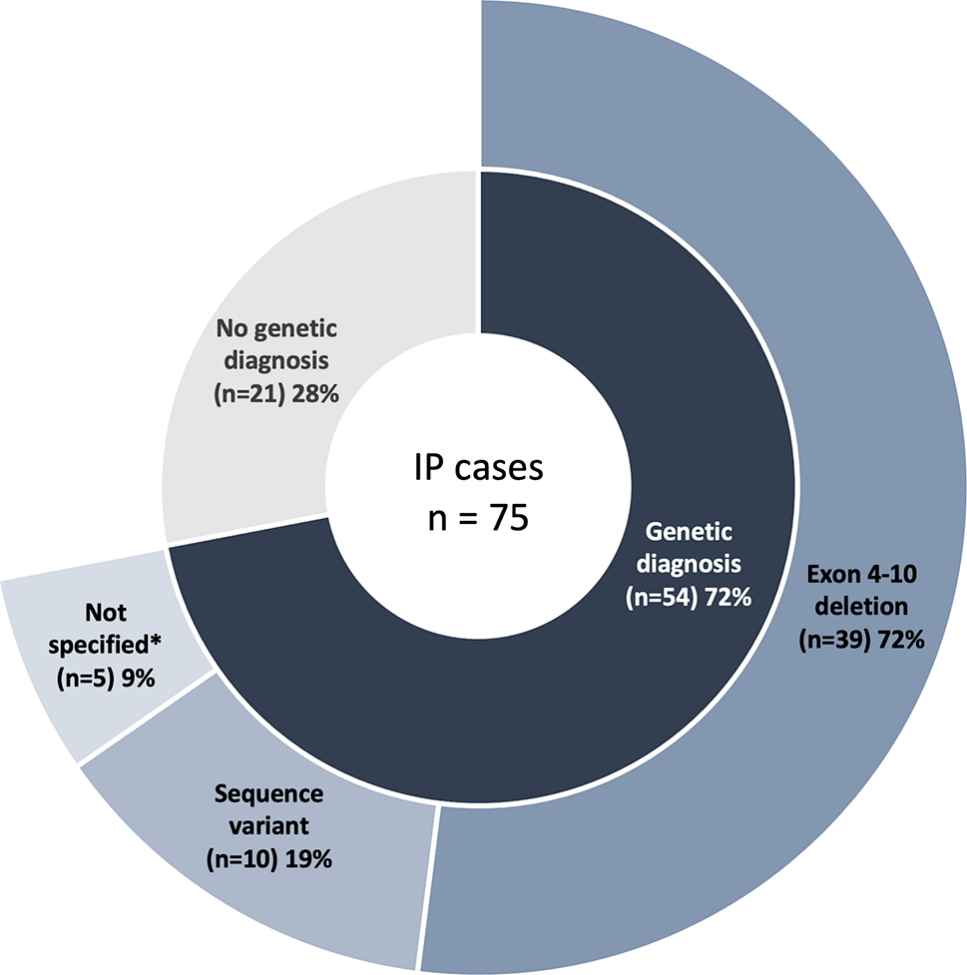
Genetic findings in the Danish IP cohort. *Details on the type of genetic variant were not available in the patient record

A total of 54 (72%) of the IP cohort were genetically confirmed (Table 1; Fig. 3), with the highest percentage among cases born after 1995 (80%). Of genetically confirmed cases, 39 (72.2%) had the recurrent deletion of exon 4–10 in *IKBKG*. Ten had point mutations including frameshift, nonsense, and splice-site variants; of these, four had variants in exon 4, three in exon 10, and the remaining variants were intronic or exon 3 variants. Five cases were described as genetically confirmed but without the variant type specified. Genetic diagnosis was followed by referral to genetic counseling in 28 (51.9%).

Over half (40, 53.3%) of the identified cases had a positive family history of either verified IP disease and/or IP-related phenotypic findings in female relatives, while the remaining cases were described as isolated (Table 1). All four male patients had presentation of IP that suggested mosaicism (skin lesions involving only parts of the body); however, fewer than three male cases were confirmed by genetic analysis of peripheral blood.

### Clinical characteristics

The median age at diagnosis was 3 years (IQR: 0–29 years) for the total cohort and 3 weeks (IQR 1–11 weeks) for cases born within the study period (incident cohort). The clinical characteristics of the total cohort are shown in Table 1. We identified 23 (30.7%) patients diagnosed with central nervous system (CNS) involvement and 17 (22.6%) with ophthalmologic involvement. The most frequent CNS disorders were seizures (*n* = 10, 13.3%) and spastic paresis/motor impairment (*n* = 7, 9.3%). The most frequent ophthalmologic manifestations were retinal vasculopathy (*n* = 11, 14.7%) and visual loss (*n* = 9, 12%). Only five patients (6.7%) did not have skin involvement reported in their medical records (Table 1). Other ectodermal derivatives were also commonly affected, including hair (*n* = 20; 26.7%), teeth (*n* = 44; 58.7%), and nails (*n* = 12; 16%).

In several patients, we noted a history of atopic disorders (*n* = 15; 20%), miscarriages (*n* = 11; 15.5% of females), and recurrent infections (*n* = 9; 12%).

## Discussion

In this study, we report a birth prevalence of IP of 2.37 (95% CI: 1.74–3.25) per 100,000 from 1995 to 2020 based on a nationwide validated cohort of IP patients in Denmark. Furthermore, we provide clinical and genetic characteristics of the cohort.

The key strength of this study was the population-based nationwide design with the inclusion of several data sources, increasing the chance of case detection. Furthermore, we obtained a high validation rate (91%) and a detailed patient characterization from the medical records. However, some limitations should be noted. First, older patients and mild cases without a hospital diagnosis record may have been missed. Such incompleteness may have resulted in an underestimate of the IP prevalence and overestimates of the prevalence of more severe manifestations. However, the estimates of various clinical data may also have been underestimated as they are based on information documented in the patient’s medical records rather than systematic clinical evaluation. Also, we were unable to describe the chronology of different manifestations in patients in our study material. Finally, even with our nationwide design, the cohort remains relatively small due to the rare nature of the disease.

We found a point prevalence of 1.21 per 100,000 and a birth prevalence of 2.37 per 100,000 live births from 1995 to 2020. Because our design carries the aforementioned risk of missing some older IP cases, we believe our birth prevalence estimate is closer to the true prevalence than our point prevalence. Our birth prevalence calculations covered birth cohorts 1995–2020, and allowed a diagnostic delay until at least six months of age for all. Although we may have missed some cases diagnosed at higher ages in the most recent calendar years, this likely had little impact on estimates considering that most cases in the incident cohort IP were diagnosed within the first 11 weeks of life. This early clinical presentation of IP is in line with previous studies [3].

Our prevalence estimates are generally higher than previous estimates. Our birth prevalence of 1 per 42,111 (including both male and female births) is almost twice as high as the birth prevalence estimate from the Orphanet report of 1.2 per 100,000 births (equals 1:83,333) [16, 22]. This estimate was based on 29 cases of IP among 2,401,473 births from 16 EUROCAT registries and likely represents an underestimate due to incomplete reporting [22]. A recent population-based Swedish study using data from a nationwide hospital registry reported a 5-year period prevalence of 1 per 41,000 [17]. However, the lack of case validation may have caused some overestimation considering the positive predictive value of 73% for an IP diagnosis in our study. The discrepancies in prevalence estimates emphasize the importance of complete registration of IP patients, stringent case validation, and transparency of methods used to compute estimates when using registry data to study the disease prevalence.

Our study confirms recurrent deletion of exon 4–10 in *IKBKG* as the main cause of IP [23]. We found a 1:9 male-female ratio in our cohort, comparable to previous studies [4]. We noted a higher proportion of familial cases (53%) in our cohort compared with previous studies (approximately 25–30%) [1, 3, 24, 25]. Importantly, the proportion of genetically confirmed cases among cases tested increased from 84 to 93%, when considering only cases born within the study period, suggesting improved diagnostic yield in recent years.

Several cohorts of IP patients have been described in the literature, providing frequencies of the clinical features of patients [1, 6, 24, 26, 27]. To our knowledge, there are no population-based studies of IP characteristics. The prevalence of CNS involvement has been reported to be approximately 30% [1, 24, 28], similar to our estimate. Ocular involvement has been reported in varying frequencies (20–70%), likely influenced by patient selection and the extent of ophthalmologic examination [24, 25, 29, 30]. Ocular involvement in 23% of patients in our cohort is thus in the lower range; however, we emphasize that this may be at least partly explained by the lack of systematic patient evaluation.

The various phenotypical features and comorbidities in IP, including a history of miscarriages in 15%, indicate that IP patients may experience significant morbidity with a profound impact on patients’ lives. The miscarriage risk of male fetuses of female IP patients is well-known due to the lethal effect of *IKBKG* deletions in males. Since we did not have a comparison group for this study, it is difficult to quantify the possible increased risk of atopy and infections associated with IP. Of notice, *IKBKG* is involved in immune regulation, and single case reports suggest that patients with IP have a risk of immunodeficiency with impaired NF-kappa-B signaling due to non-skewed X-inactivation [31–35].

## Conclusions

In conclusion, we identified and characterized a nationwide population-based cohort of 75 IP patients and estimated a population-based birth prevalence of 2.37 per 100,000 live births, which was twice as high as previously estimated.

## Data Availability

The data that support the findings of this study are not available due to ethical considerations. According to the Danish Act on Processing of Personal Data, private and public institutions may obtain the health data used in the current study after obtaining the necessary project-specific approvals. The data from the Danish National Patient Registry used to identify the study cohort was obtained from the Danish Health Data Authority: https://sundhedsdatastyrelsen.dk/da/english.

## Abbreviations

CI: Confidence Interval
CNS: Central Nervous System
CPR: Unique personal identifier of Danish citizens
DNPR: Danish National Patient Registry
HED-ID: X-linked Hypohidrotic Ectodermal Dysplasia associated with Immunodeficiency
ICD: International Classification of Diseases
IKBKG/(NEMO): Inhibitor of Kappa polypeptide gene enhancer in B-cells, Kinase Gamma/Nuclear Factor kappa B, Essential Modulator
IP: Incontinentia Pigmenti
IQR: Interquartile Range
OMIM: Online Mendelian Inheritance in Man
RAREDIS: Danish National Database of Rare Genetic Diseases
STROBE: STrengthening the Reporting of OBservational studies in Epidemiology
